# Association between marijuana use and erectile dysfunction in a sample of sexual minority men: a cross-sectional analysis

**DOI:** 10.1186/s42238-026-00411-1

**Published:** 2026-02-17

**Authors:** Marion Otieno, Yunan Zhao, Alvin Tran

**Affiliations:** 1 University of New Haven, 300 Boston Post Rd, West Haven, CT 06516 USA; 2 New York University, 227 E 30Th St, New York, NY 10016 USA

## Abstract

**Background:**

Erectile dysfunction (ED) is a condition characterized by difficulty in achieving or maintaining an erection, consequently affecting sexual performance. ED is often a symptom of underlying health conditions such as cardiovascular heart disease and can lead to psychological distress. Previous studies show an association between marijuana use and ED through its potential impact on the endocannabinoid pathways. However, few studies have explored this relationship among sexual minority men (SMM). Considering this knowledge gap, this study aims to assess the association between marijuana use and erectile dysfunction in a sample of SMM in the United States.

**Methods:**

A secondary analysis of the Men’s Body Project (MBP)—a cross-sectional study involving an online survey assessing SMM health outcomes—data was conducted. Multivariable logistic regression and bivariate analyses were used to examine the association between marijuana use and ED. Participants provided demographic information, self-reported ED, erection confidence, erection difficulty, and history of substance use.

**Results:**

A total of 549 participants completed the survey (52.1% gay and 47.9% bisexual). The prevalence of ED was higher in bisexual men compared to gay men. Results from the adjusted logistic regression analysis suggested that people who use marijuana had 1.83 times the odds (95% CI: 1.23, 2.74) of reporting erectile dysfunction compared to non-users.

**Conclusion:**

Our results suggest a significant association between marijuana use and elevated odds of experiencing erectile dysfunction. These findings highlight the importance of integrating sexual health screening into primary care and considering substance use during sexual health assessments to support early identification and management of ED. Additional research is needed to explore temporal and causal relationships between marijuana use frequency and erectile function.

## Introduction

Erectile dysfunction (ED) is a major sexual health concern among men, and is a condition characterized by difficulty in achieving or maintaining an erection, consequently affecting sexual performance (NIH 1993). Data from the 2021 National Survey of Sexual Wellbeing estimates the prevalence of erectile dysfunction in the United States (U.S.) to be approximately 24% (Mark et al. 2024), with prevalence rates increasing with age (Mark et al. 2024; MacGill 2024). Aside from affecting one’s sexual performance, ED is linked to a number of adverse outcomes, such as stress, relationship problems, and issues with reduced self-confidence (Leslie and Sooriyamoorthy 2025). Furthermore, ED is often a symptom of underlying physical or psychological health conditions such as heart disease, diabetes, obesity, atherosclerosis, anxiety, and depression (Mayo Clinic 2025).

Male sexual arousal is a multifaceted process involving the nervous, endocrine, and vascular systems (Institute and of Diabetes and Digestive and Kidney Diseases 2025). While physical and psychological conditions are established contributing factors to ED that affect these systems (Mayo Clinic 2025; Institute and of Diabetes and Digestive and Kidney Diseases 2025), lifestyle factors, such as substance use, have been shown to play a significant role (Institute and of Diabetes and Digestive and Kidney Diseases 2025). For instance, the use of tobacco is reported to hamper the nitric oxide transduction pathway, particularly the neuronal and endothelial nitric oxide isoforms, responsible for smooth muscle relaxation to enable arterial blood flow into the penile corpus cavernosum (Kovac et al. 2015). Unlike tobacco, however, which has well-documented effects on erectile function, the impact of using marijuana on male sexual health, particularly around experiences with ED, is inconclusive and has not been fully explored (Pizzol et al. 2019; Kilmer 2017; Shamloul and Bella 2011).

The use and legalization of marijuana, though contentious, has become mainstream globally. In the U.S., marijuana has been legalized for recreational use in at least 24 states, whereas other states maintain varying policies, including illegality, decriminalization, or medical-only use (Bryan 2024). Scientific evidence backs the therapeutic effects of marijuana, including its effectiveness as an antiemetic in chemotherapy-induced vomiting, relief from chronic pain, and improved spasticity in multiple sclerosis patients (National Academies of Sciences, Engineering, and Medicine 2017). Among recreational users, a commonly cited reason for marijuana use is its perceived aphrodisiac effect (Acharya et al. 2025; Ghadigaonkar and Murthy 2019). Erectile function is hypothesized to be influenced, in part, by the endocannabinoid system through the activation of cannabinoid receptors (Ghadigaonkar and Murthy 2019). Cannabinoid receptors are proteins expressed throughout the body that respond to endogenous cannabinoids as well as exogenous cannabinoids, such as those found in marijuana. Preclinical studies suggest that cannabinoid receptors located in the hypothalamus and in penile tissue, including the corpus cavernosum, may influence erectile function through central and peripheral mechanisms, respectively (Ghadigaonkar and Murthy 2019; Gratzke et al. 2010). Marijuana may influence erectile function indirectly by alleviating symptoms of anxiety or depression (Pizzol et al. 2019), which are established psychological contributors to ED. Preclinical evidence suggests that activation of cannabinoid receptors in penile tissue by endogenous cannabinoids can induce smooth muscle relaxation and vasodilation, thereby increasing penile blood flow, a key physiological requirement for achieving and maintaining an erection (Acharya et al. 2025). However, chronic exposure to the primary psychoactive constituent of marijuana, Δ9-tetrahydrocannabinol (THC), has been shown in experimental models to dysregulate endocannabinoid signaling pathways, which may adversely affect erectile function over time (Acharya et al. 2025; Ghadigaonkar and Murthy 2019). Disruption of endocannabinoid signaling pathways may also be associated with reduced libido (Acharya et al. 2025), which can contribute to erectile dysfunction. Emerging evidence further suggests that cannabinoid exposure may be associated with decreased testosterone levels (Acharya et al. 2025; Ghadigaonkar and Murthy 2019), and alterations in sperm quality (Acharya et al. 2025). Although these findings raise concerns regarding potential effects on male reproductive health and fertility, additional research is needed to clarify the relationship between marijuana use and erectile dysfunction.

Concerns regarding marijuana’s potential impact on erectile function are further informed by findings from comparative studies. An analysis of mainstream and sidestream smoke demonstrated that marijuana smoke contains several of the same toxic constituents found in tobacco smoke (Moir et al. 2008). These similarities suggest that marijuana smoke may affect biological pathways implicated in erectile function in ways comparable to tobacco exposure. Furthermore, evidence from epidemiologic studies remains mixed. A systematic review and meta-analysis reported a higher prevalence of erectile dysfunction among individuals who use marijuana (69.1%) compared with non-users (34.7%) (Pizzol et al. 2019). Similarly, in a study by Kumsar et al. (2016) examining sexual dysfunction among men with substance use disorders, 35% of people who use marijuana self-reported severe erectile dysfunction. In contrast, other studies have found no significant association between marijuana use and erectile dysfunction (Smith et al. 2010).

Given the inconsistent findings in the existing literature, this study aimed to examine the association between marijuana use and erectile dysfunction among sexual minority men (SMM) in the U.S. Sexual minority men present unique considerations in this area of research, as much of the existing literature has focused primarily on men in the general population. National data from the 2023 Substance Abuse and Mental Health Services Administration indicate that marijuana use is nearly twice as prevalent among SMM compared to straight males (Substance Abuse and Mental Health Services Administration 2024). This disparity may be understood through the minority stress theory(Frost and Meyer 2023), which posits that sexual minority individuals experience chronic stressors related to stigma, discrimination, and social marginalization in addition to general life stressors. These stressors may contribute to adverse health outcomes through increased psychological distress and the use of maladaptive coping strategies, including substance use, which may in turn affect sexual function. Accordingly, we hypothesized that marijuana use would be associated with increased odds of erectile dysfunction in this population.

## Methods

### Participants

The present study utilized existing data from the Men’s Body Project (MBP), a cross-sectional online survey conducted among bisexual and gay men residing in the U.S. Participants were recruited between March and May 2020 through Qualtrics Survey Panels, an online research platform that maintains diverse participant panels and employs multiple quality control procedures. Panel members were invited to complete an anonymous online survey and were compensated by Qualtrics using cash, airline miles, vouchers, or other incentives, with compensation determined by factors such as survey length and panelist profile.

To enhance data quality and reduce the likelihood of fraudulent or automated responses, Qualtrics implemented standard response screening procedures, including panel authentication, duplicate response checks, and attention and consistency checks. Eligible participants were those who were aged 18–50 years, identified as cisgender gay or bisexual men, resided in the U.S., and were able to complete the survey in English. All participants provided informed consent prior to participation. The study protocol was approved by the Institutional Review Board of the University of New Haven (#2020–015). Additional details about the MBP have been published elsewhere (Tran et al. 2023a, 2023b).

### Measures

Demographic variables: Participants reported their age (in years), race, ethnicity, sexual orientation, employment status, relationship status, body mass index (BMI, kg/m^2^), and smoking status in the past 12 months (yes/no).

Marijuana use: Marijuana use was assessed via one item: In the past 12 months, how often did you use marijuana? Response options ranged from never, less than once a month, 2–3 times/month, 1–2 times/week, 3–5 times/week, to 6 or more times/week. A binary variable (marijuana use vs. no use) was created to assess the association between annual marijuana use and erectile dysfunction.

Erectile dysfunction: We assessed participants’ experiences with erectile dysfunction using three items from the Growing Up Today Study (Growing Up Today Study 2013). During the past 12 months, how would you rate your confidence that you could get and keep an erection? Response options included “very low,” “low,” “moderate,” “high,” and “very high.”

In the past 12 months, during sexual activity, how difficult was it to maintain your erection to completion? Response options ranged from “not difficult,” “slightly difficult,” “difficult,” “very difficult,” to “extremely difficult.”

Do you consider that you have problems achieving or maintaining an erection? Response options included “yes” or “no.”

### Statistical analyses

Descriptive statistics, including frequencies and percentages, were calculated for demographic variables, erection confidence, and erection difficulty. The distribution of these variables was compared between people who use marijuana and non-users using chi-square tests. These tests evaluated the differences between users and non-users of marijuana. The association between marijuana use and erectile dysfunction was examined using multivariable logistic regression models. The adjusted model controlled for demographic characteristics, including age, race, ethnicity, sexual orientation, relationship status, and employment status. We reported odds ratios (ORs) and their 95% confidence intervals for both unadjusted and adjusted models, representing results without and with demographic adjustments. Statistical significance for all analyses was set at p values < 0.05.

## Results

Table 1 describes the sociodemographic characteristics of the study sample. A total of 549 men responded to the survey and were included in the analyses. Of the 549 participants, 52.1% (*n* = 286) identified as gay and 47.9% as bisexual (*n* = 263). The majority of the participants identified as White, consisting nearly 72.0% of the total sample (*n* = 392), followed by Black (*n* = 76, 13.8%) Asian/Pacific Islander (*n* = 44, 8.0%), and American Indian/other (*n* = 37, 6.7%). Close to 19.0% of the sample was Hispanic. Additionally, most participants were aged 35−50 years (54.6%), followed by those between 25–34 years (25.9%) and 18–24 years (19.5%). More than half of the people who use marijuana (52.3%) were bisexual, compared to their gay counterparts (47.7%). Regarding smoking behavior, people who use marijuana were more likely to report smoking in the past 12 months (61.2%) compared to non-users (25.6%). Smoking was equally significantly associated with marijuana use (*p* < 0.001).

**Table 1:** Sociodemographic characteristics of people who use marijuana compared to non-users (*N* = 549)

	**People Who Use Marijuana (*N* = 237)**	**Non-Users (*N* = 312)**	**Overall (*N* = 549)**	***p*-value**
Age (years)				< 0.001*
18–24	51 (21.5%)	56 (17.9%)	107 (19.5%)	
25–34	83 (35.0%)	59 (18.9%)	142 (25.9%)	
35–50	103 (43.5%)	197 (63.1%)	300 (54.6%)	
Ethnicity				0.120
Hispanic	52 (21.9%)	51 (16.3%)	103 (18.8%)	
Non-Hispanic	185 (78.1%)	261 (83.7%)	446 (81.2%)	
Race				0.015*
White	162 (68.4%)	230 (73.7%)	392 (71.4%)	
Black	43 (18.1%)	33 (10.6%)	76 (13.8%)	
Asian/Pacific Islander	13 (5.5%)	31 (9.9%)	44 (8.0%)	
American Indian/Other	19 (8.0%)	18 (5.8%)	37 (6.7%)	
BMI (kg/m2)				0.472
< 18.5	11 (4.6%)	11 (3.5%)	22 (4.0%)	
18.5 − 24.9	102 (43.0%)	117 (37.5%)	219 (39.9%)	
25.0 − 29.9	77 (32.5%)	114 (36.5%)	191 (34.8%)	
≥ 30.0	47 (19.8%)	70 (22.4%)	117 (21.3%)	
Sexual Orientation				0.086
Gay	113 (47.7%)	173 (55.4%)	286 (52.1%)	
Bisexual	124 (52.3%)	139 (44.6%)	263 (47.9%)	
Relationship Status				0.568
Single/Dating	125 (52.7%)	182 (58.3%)	307 (55.9%)	
Living with a partner	35 (14.8%)	45 (14.4%)	80 (14.6%)	
Married/engaged	65 (27.4%)	72 (23.1%)	137 (25.0%)	
Divorced/Widowed/Separated/Other	12 (5.1%)	13 (4.2%)	25 (4.6%)	
Employment Status				0.511
Full-time	149 (62.9%)	98 (63.5%)	347 (63.2%)	
Part-time	23 (9.7%)	26 (8.3%)	49 (8.9%)	
Student	21 (8.9%)	30 (9.6%)	51 (9.3%)	
Unemployed	31 (13.1%)	49 (15.7%)	80 (14.6%)	
Other	13 (5.5%)	9 (2.9%)	22 (4.0%)	
Smoke (past 12 months)				< 0.001*
Yes	145 (61.2%)	80 (25.6%)	225 (41.0%)	
No	92 (38.8%)	232 (74.4%)	324 (59.0%)	
Erection Confidence				0.277
Very low	9 (3.8%)	16 (5.1%)	25 (4.6%)	
Low	25 (10.5%)	20 (6.4%)	45 (8.2%)	
Moderate	53 (22.4%)	86 (27.6%)	139 (25.3%)	
High	62 (26.2%)	74 (23.7%)	136 (24.8%)	
Very High	88 (37.1%)	116 (37.2%)	204 (37.2%)	
Erection Difficulty				0.090
Extremely difficult	20 (8.4%)	20 (6.4%)	40 (7.3%)	
Very difficult	22 (9.3%)	15 (4.8%)	37 (6.7%)	
Difficult	27 (11.4%)	26 (8.3%)	53 (9.7%)	
Slightly difficult	55 (23.2%)	75 (24.0%)	130 (23.7%)	
Not difficult	113 (47.7%)	176 (56.4%)	289 (52.6%)	

*Indicates a *p*-value < 0.05. Chi-Square (χ2) tests and student’s t-test were performed for categorical variables and continuous variables, respectively to examine differences between

people who use marijuana and non-users

Table 2 represents results for both the unadjusted and adjusted logistic regression models for erectile dysfunction in people who use marijuana. The unadjusted results showed that people who use marijuana had 1.65 times the odds (95% CI: 1.14, 2.39) of reporting ED compared to non-users. Even after adjusting for demographic characteristics and other potential confounders, the association remained significant with increased odds, indicating that people who use marijuana had 1.83 times the odds (95% CI: 1.23, 2.74) of reporting erectile dysfunction compared to non-users. However, Chi-square (χ^2^) tests suggested that erection confidence and erection difficulty were not significantly associated with marijuana use (*p* > 0.05).

**Table 2:** Odds of erectile dysfunction among people who use marijuana compared to non-users (*N* = 549)

	Unadjusted		Adjusted^a^	
	Odds Ratio [95% CI]	p value	Odds Ratio [95% CI]	*p* value
Erectile Dysfunction	1.65 [1.14, 2.39]	0.003*	1.83 [1.23, 2.74]	0.003*

Reference group: non-users

*Indicates a *p*-value < 0.05

^a^Adjusted for demographic characteristics include age, race, ethnicity, sexual orientation, relationship status, and employment status

## Discussion

Our study examined the association between marijuana use and erectile dysfunction in a sample of SMM in the U.S. Results from logistic regression analyses indicated a significant association between marijuana use and higher odds of reporting erectile dysfunction, even after adjusting for demographic characteristics.

These findings are broadly consistent with a systematic review and meta-analysis (Pizzol et al. 2019), which reported a higher prevalence of erectile dysfunction among individuals who use marijuana (69.1%) compared with non-users (34.7%) and an odds ratio of 3.83 (95% CI: 1.30–11.28). However, differences between the prevalence estimates reported by Pizzol et al. (2019) and those observed in the present study should be interpreted considering important methodological differences. The meta-analysis included heterogeneous samples of men drawn from multiple observational study designs and used varying assessments of erectile dysfunction, whereas the present study focused specifically on sexual minority men and employed ED measures adapted from the Growing Up Today Study. These differences in study populations and outcome measurement may have contributed to the higher prevalence estimates reported in prior studies.

Although multivariable analyses in our study suggest an association between marijuana use and erectile dysfunction, bivariate analyses did not show significant associations with erection confidence or erection difficulty. Erection confidence and erection difficulty capture specific dimensions of erectile function during sexual activity, whereas the erectile dysfunction outcome reflects a broader, self-identified clinical concern, which may partly explain why associations emerged in adjusted models but not in bivariate analyses. This discrepancy suggests that the observed association may be influenced by confounding factors accounted for in adjusted models and highlights the complexity of the relationship between marijuana use and erectile function. Taken together, these findings underscore the need for cautious interpretation and further research to clarify underlying mechanisms, including potential involvement of endocannabinoid pathways (Acharya et al. 2025; Ghadigaonkar and Murthy 2019).

Furthermore, consistent with the framework of the minority stress theory, prior studies have documented higher marijuana use among SMM (Bancroft et al. 2005), often as a coping response to chronic stressors, such as stigma (Buckner et al. 2023; Mereish 2019). Substance use has been shown to be particularly prevalent among bisexual individuals compared to gay individuals (Demant et al. 2017; Schofield et al. 2023), a pattern reflected in our study findings, where more than half of participants who reported marijuana use identified as bisexual men. Similarly, the prevalence of erectile dysfunction was higher among bisexual men than gay men in our sample. These findings suggest that bisexual men may experience a higher burden of erectile dysfunction, potentially related to differences in substance use behaviors and stress exposure. Comparative studies further support elevated rates of sexual dysfunction among sexual minority men, with evidence indicating higher odds of erectile dysfunction among homosexual men compared to heterosexual men (Barbonetti et al. 2019), and reports that approximately 45% of Belgian men who have sex with men have experienced some degree of erectile dysfunction (Vansintejan et al. 2013).

Taken together, this evidence suggests a complex relationship between marijuana use and erectile dysfunction. Substance use related to minority stress, such as stigma or discrimination, may be associated with barriers to health care access, potentially exacerbating ED prevalence. Furthermore, few studies account for the dose–response relationship, revealing critical knowledge gaps in the association between marijuana use frequency and duration, and ED. Chu et al., (Chu et al. 2023) note that a growing number of people who use marijuana may be exposed to tobacco through co-use, concurrent use, or mixing. From our findings, close to 63% of the total number of people who use marijuana reported having smoked tobacco in the past 12 months. This complicates the direct attribution of the association of ED to marijuana alone, given that tobacco use is a risk factor in ED.

This study adds to the existing body of literature on marijuana use and erectile dysfunction, particularly among a group with limited studies in this research area: sexual minority men. While our findings report an association between marijuana use and higher odds of reporting ED, the cross-sectional design of the study does not permit causal interpretation. Our study underscores the need for more health education and awareness of the potential risks of marijuana to sexual health, given its growing legalization. Even though existing literature report mixed findings, suggesting dual effects (both therapeutic and adverse effects) of marijuana on erectile function, well-designed longitudinal and experimental studies are needed to clarify causality, confounders, and dose–response relationships.

### Limitations of study

This study has several limitations related to study design and data collection that should be considered when interpreting the findings. First, erectile dysfunction was assessed using self-reported measures, which may be subject to reporting or recall bias. Second, the Men’s Body Project dataset did not capture detailed information on marijuana dosage or mode of administration, such as smoking, vaping, or edible use. This limitation is important because smoking and vaping are associated with adverse vascular effects that may independently influence erectile function (Hartford Hospital 2025). Consequently, it remains unclear whether different methods of marijuana use are differentially associated with experiences of erectile dysfunction. In addition, marijuana use was dichotomized (any use vs. no use), which precluded assessment of potential dose–response relationships between marijuana use frequency and erectile dysfunction.

In addition, the cross-sectional design of the study precludes assessment of temporal sequencing and causal inference. It is possible that erectile dysfunction preceded marijuana use or resulted from unmeasured underlying physical or psychological conditions. Reverse causation is also plausible, whereby individuals experiencing erectile dysfunction may use marijuana as a coping strategy for psychological distress or sexual performance concerns. Longitudinal studies that assess marijuana use patterns, mode of administration, and erectile function over time are therefore needed to better clarify the directionality and nature of this association.

## Conclusion

Limited studies have accounted for the association of marijuana use with erectile dysfunction in sexual minority men despite their higher substance use behaviors. The observed associations should, however, be interpreted cautiously as the cross-sectional design of this study precludes causal inference. The discrepancies in both our findings and the existing literature emphasize the need for additional studies to determine causality and account for the dose–response relationship between marijuana use frequency and erectile function. In conclusion, our findings highlight the importance of considering targeted public health interventions to address substance use behaviors and the integration of sexual health screening into primary care for SMM, particularly among bisexual men, who exhibited a higher prevalence of ED in this sample.

## Data Availability

The datasets used and/or analyzed during the current study are available from the corresponding author upon reasonable request.

## Abbreviations

SMM: Sexual minority men
ED: Erectile dysfunction
MBP: Men’s Body Project
